# Clinical evaluation of neodymium-iron-boron (Ne2Fe14B) rare earth magnets in the treatment of mid line diastemas

**DOI:** 10.4317/jced.52352

**Published:** 2016-04-01

**Authors:** Mandava Prasad, Mitta Manoj-Kumar, Singaraju Gowri-Sankar, Nellore Chaitanya, Ganugapanta Vivek-Reddy, Nettam Venkatesh

**Affiliations:** 1 Professor and Head, Orthodontics, Narayana Dental College and Hospital, Nellore, AP, India; 2 Postgraduate student, Orthodontics, Narayana Dental College and Hospital, Nellore, AP, India; 3 Professor, Orthodontics, Narayana Dental College and Hospital, Nellore, AP, India; 4 Senior lecturer, Orthodontics, Narayana Dental College and Hospital, Nellore, AP, India

## Abstract

**Background:**

To evaluate the closure of midline diastema using the Neodymium-Iron-Boron magnets and to compare the treatment duration of midline diastemas with the use of magnets compared to regular orthodontic treatment.

**Material and Methods:**

Thirty patients with age group 12 to 30 years with the midline diastema ranging from 0.5 to 3mm were selected. These patients were divided into two groups. Diastema closure in one group was accomplished by conventional method, in other group was done with Ne2Fe14B magnets. These magnets were fitted to the labial surfaces of the maxillary central incisors such a way that the opposite poles of the magnets face each other. At each appointment, study models and radiographs were taken for study subjects and the midline diastema was measured using digital vernier calipers on the study models obtained. Descriptive statistics carried out using *Paired t-test*.

**Results:**

Subjects treated with Ne2Fe14B magnets showed a significant difference compared to fixed orthodontic appliance subjects with respect to time of closure, rate of space closure and incisal inclination. Significant difference between 2 groups with reduction of 64.6 days in time to diastema closure in subjects treated with Ne2Fe14B magnets (*P*<0.05).

**Conclusions:**

Ne2Fe14B magnets more efficient in complete closure of mid line diastema in less duration of time.

** Key words:**Midline diastema, Ne2Fe14B magnets, rare earth magnets, space closure.

## Introduction

Orthodontics is a dynamically growing science. It is constantly undergoing development and is evolving through the discovery of newer techniques and improvements over the older ones. The improvement of facial and dental esthetics has rapidly become one of the desirable objectives of orthodontic treatment (1). Angle (1907) described the dental midline diastema as a rather common form of incomplete occlusion characterized by a space between the maxillary and less frequent mandibular central incisors. Broadbent described the normal closure of this space by the medial erupting path of the maxillary lateral incisors & canines. It may be persistent in some individuals due to abnormal labial frenum, oral habits, muscular imbalances, inter-maxillary osseous cleft, missing anterior teeth, periodontal disease with bone loss, generalized microdontia, maxillary pathologies, mesiodens, etc (1).

Midline diastema is frequently cited as a malocclusion with high relapse incidence. A number of treatment modalities has been explained in different literatures. Among them, the present study considered the neodymium-iron-boron (Ne2Fe14B) rare earth magnets as a mode in the closure of midline diastema. Magnets (from Greek μαγνήτης λίθος, “Magnesian stone”) is a material or object that produces a magnetic field. This invisible field pulls on nearby magnetic materials or repels other magnets based up on unlike and like poles. Magnetic forces can be used to generate the force for tooth movement and orthopaedic treatment. Advantages of magnetic force delivery include good force control at short distances, no friction, and no material fatigue (2). Correct identification of a patients’ arch form is an important aspect of achieving a stable, functional and aesthetic orthodontic treatment result (3). Magnets in dentistry were ﬁrst used to improve retention of dentures and Maxillofacial prostheses. The repulsive force of magnets was utilized to keep the upper and lower complete dentures in place, while attractive forces to hold the prostheses to the dentoalveolar segment. The earlier magnets were made of either aluminum-nickel-cobalt (AlNiCo) or platinum-cobalt (Pt Co) alloy. 

The force magnitudes obtained are dependent on the distance between two magnets. Force levels would decay dramatically with the separation of repulsive magnets and would increase as the distance between attractive magnets is reduced (4). The use of AlNiCo and PtCo magnets in dentistry was limited due to the dimensions of the magnet, which are further increased by the coating material used to prevent corrosion in the oral cavity. The introduction of rare earth magnets, especially Samarium-Cobalt (SmCo) and Neodymium-Iron-Boron (NdFeB) magnets between 1970 and 1980, revived interest in their use in prosthodontics and orthodontics specialties. Traditional force delivery systems in orthodontics include the use of wires, springs and elastics. Metallic springs and multiple elastomers cause mechanical forces which deteriorate to levels that no longer induce tooth movement (Hooke’s law) unlike magnetic forces that can cause constant tooth movement. Advantages of magnetic force delivery include minimal tooth tipping, less chair time, no further activations, maintainable oral hygiene, cheaper and recyclable, no friction, and no material fatigue (5).

The magnets initially used were bulky and had toxic effects. However, the current available literature evaluating magnetic fields shows no evidence of any direct or indirect toxic effects. Improved safety with better coating on rare earth magnets led to a dramatic reduction in magnet size stimulated further interest in the field of orthodontics (6). Considering epidemiologically high incidence of midline diastemas in our institution and also the socio-economic status of the population, it was worthwhile to treat these cases with a cheaper and efficient method using magnets. Conventional Orthodontic treatment is the most expensive of all dental treatments and also not affordable by a large number of Indian populations. Owing to the low economic status of this region this technique would benefit a large number of our population. The purpose of the study is to evaluate the closure of midline diastema using the Ne2Fe14B magnets and compare its treatment duration with the non- magnetic regular fixed orthodontic appliance (Straight wire Appliance).

## Material and Methods

A prospective clinical trial method was opted. Ethical approval for the study was obtained from the institutional ethical committee. The participants and their parents were informed of the study, Written consent was received from all the subjects. The subjects were recruited with the following criteria.

The Inclusion criteria include patients less than 25 yrs of age at the start of treatment, Angles class I or II dentoalveolar malocclusion, midline diastema ranging from 0.7 -3.5 mm in permanent dentition. The Exclusion criteria were transient malocclusions, impacted or unerupted permanent teeth, deformities with cystic lesions and cleft lip and palate cases. Thirty patients (16 female, 14 male) fulfilled the inclusion criteria. Their demographics are shown: Age of the sample was shown in  Table 1, sex distribution and midline diastema scores of the sample were also recorded. The subjects were randomly allocated for treatment with rare earth Ne2Fe14B magnets and conventional fixed appliance therapy i.e. Straight wire appliance as group 1 and 2.

**Table 1:**
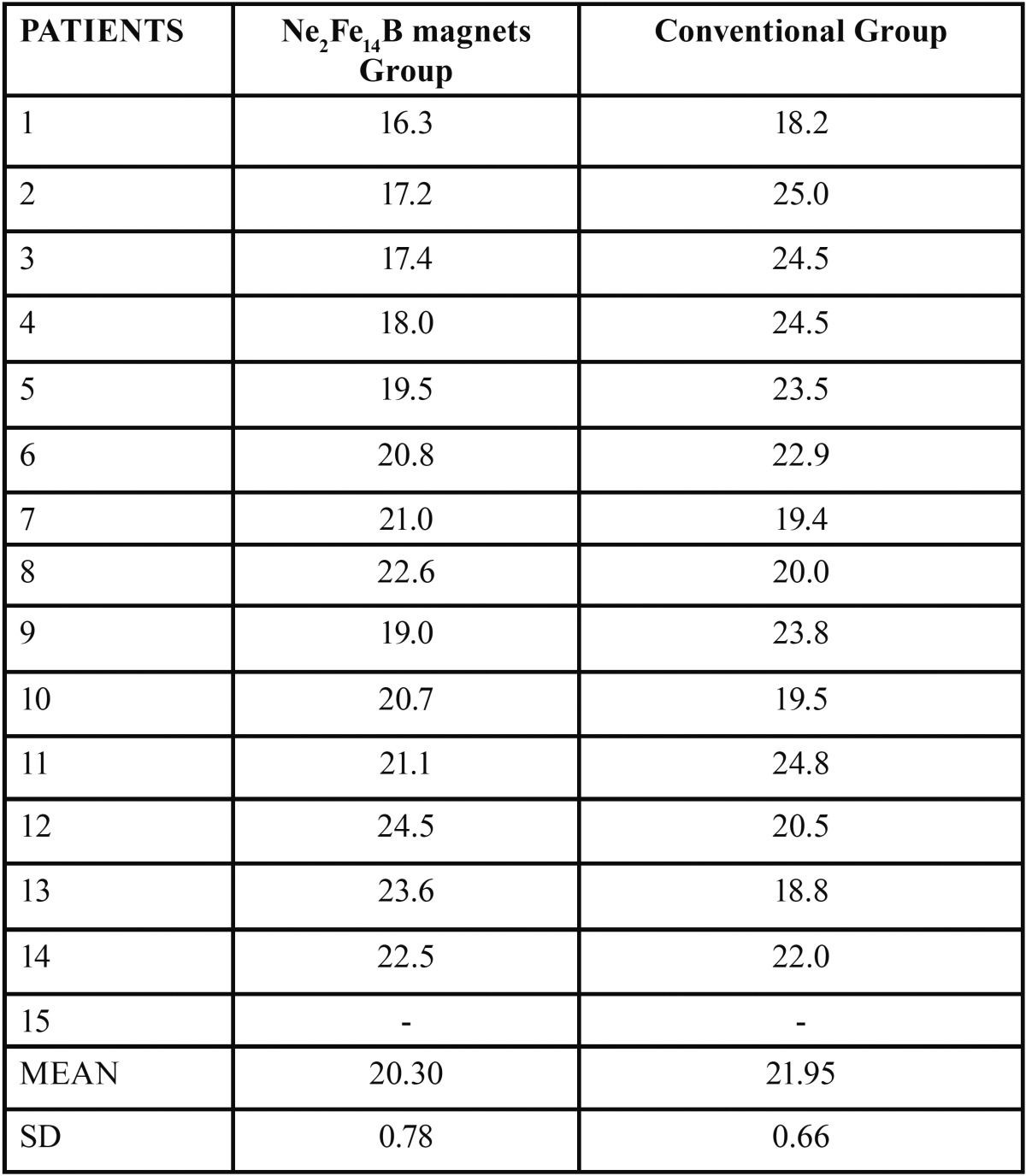
Age distribution of the sample.

**Figure 1:**
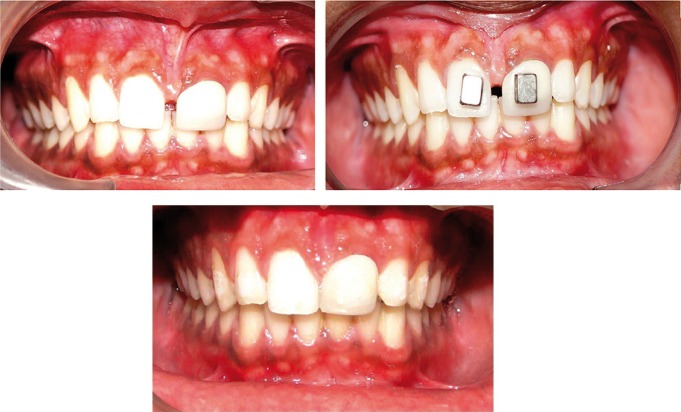
Pre and post treatment intra oral photographs with bonded magnets.

After the satisfaction of inclusion criteria and consent obtained from the subjects, the successive subjects were properly diagnosed for high frenal attachments and continued closure of midline diastema after the process of frenectomy. In the present study, rectangular Ne2Fe14B magnets (Fig. 1) were chosen. This new cobalt-free alloy had magnetic properties superior even to those of cobalt-samarium, with the energy product being as high as 341KJ/m3 (7), extremely high magnetic saturation and good resistance to demagnetization. Their excellent magnetic properties allowed the production of very small magnets (8). They are less costly to produce than Sm-Co alloys, and hence, now the main rare earth permanent magnet in use today.

Magnets 5 mm in length, 4 mm width and 3 mm in thickness were used in the study. Maximum attractive force between the magnets used in this study was between 0.94 and 1.15 N. The magnets applied 117.5grams of force. Magnets are added with Nickel - Chromium plating for tarnish and corrosion resistance. Magnets of unlike poles are placed facing each other such that the magnetic axial lines are parallel to each other. Grooves were made on the magnets for the mechanical interlocking with the composite restoration. By means of the direct bonding technique, the magnets were fixed to the labial surface of the upper two central incisors. The bracket systems used were 0.018 slot MBT (AO) for conventional fixed appliance therapy and cases with magnets on the teeth other than the incisors considering the esthetics and minimizing the space between the anterior teeth.

Bonding methods were standardized between the groups. Study models, photographs and radiographs were taken from each patient at the onset (T1) of the study. After the placement of magnets, Group 1 patients were reviewed every 3 days for measuring the change occurred. Study models, photographs and radiographs were taken from each patient at the mid period (T2) and completion (T3) of the study.

In other group, the arch wire sequence was 0.014’’, 0.016’’ and 0.016 x 0.022’’ NiTi and 0.016 x 0.022’’ SS working wires in place for 4 weeks until the wire passively engaged in all the bracket slots before proceeding to the higher wire. The patients were reviewed every 4 weeks. The primary outcome measure was the mean amount of space closure in millimeters in weekly intervals. The time for complete space closure was calculated for each patient in days.

Maxillary incisal angulation to bicondylar line in degrees & maxillary incisal inclination to maxillary plane in degrees were assessed for all the patients at T1, T3 using the angular measurement in lateral cephalograms. Each model was numbered for identi-fication. Therefore the researcher was blinded to patient’s name, the group type during data collection to minimize systematic error. The study models were measured with Digital calipers: measuring to 0.01 mm. The same researcher did all measurements.

The same author retracing assessed the method error and redigitizing 30 randomly selected cephalometric radiographs after a period of 15 days. Method error coefficients for all measurements were calculated and were within acceptable limits (range 0.98-0.99). Thus intra examiner reliability was assessed by random selection of models from the records. The cephalometric records were retraced, and the measurements of the cephalometric variables were repeated. The rate of space closure, in mms per month (4 weeks) and a 4-monthly rate, was then calculated.

Demographic and clinical characteristics were investigated with conventional descriptive statistics. Paired t tests were used for finding the test of significance for normally distributed variables. Significance of the difference in treatment time to space closure between the groups was done by Paired Student t-test. Using Dixon *et al.* (9) method assessed a prior sample size. Using this method, we estimated that a sample size of 13 subjects in each group would be sufficient to detect a difference in the rate of space closure of 3 mm (SD: 2.58) over 3 months, with a power of 90% and significance level of 0.05. To account for a 15% drop-out rate, the sample size was increased to 15 participants per group.

An analysis was also done for time to alignment as dependent variable with midline diastema closure, change in maxillary incisal inclination, and age and diastema scores as independent variables. Variables as time to alignment, space closure, and maxillary incisal inclination and midline diastema scores were correlated. Statistical analysis was performed with the SPSS 15.01 for Windows. A prospective clinical trial was done to evaluate significant longitudinal changes during T1, T2, and T3.

## Results

The mean, standard error of the mean, significance of the measurements, and the changes during T1, T2, and T3 are shown ( Table 2). 30 subjects were recruited for the study and of them 28 subjects completed the study and those who failed to complete the treatment were omitted from the statistical analysis. “The mean, standard error of the mean, significance of the measurements, and the changes during T1, T2, and T3 are shown ( Table 2).

**Table 2:**
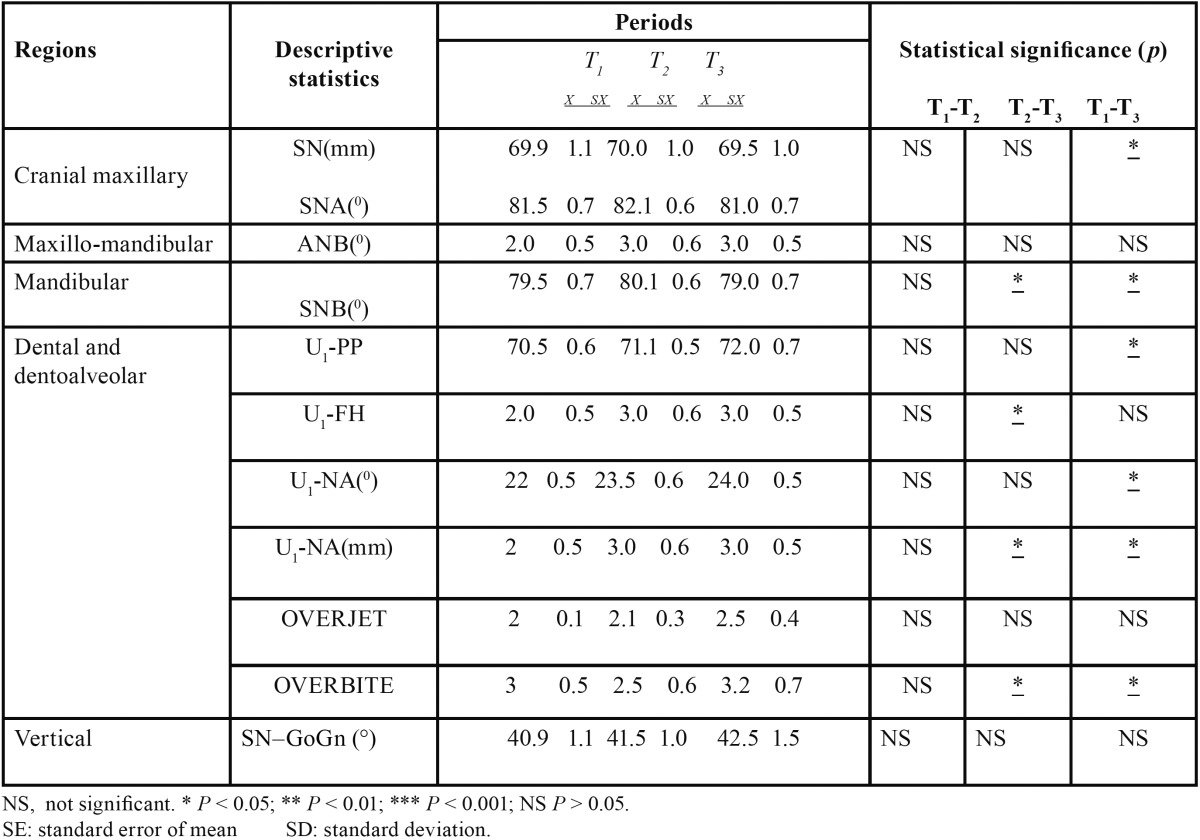
Changes in the descriptive statistics, the mean value (x), standard error (sx), and their statistical significance in the different periods.

**Figure 2:**
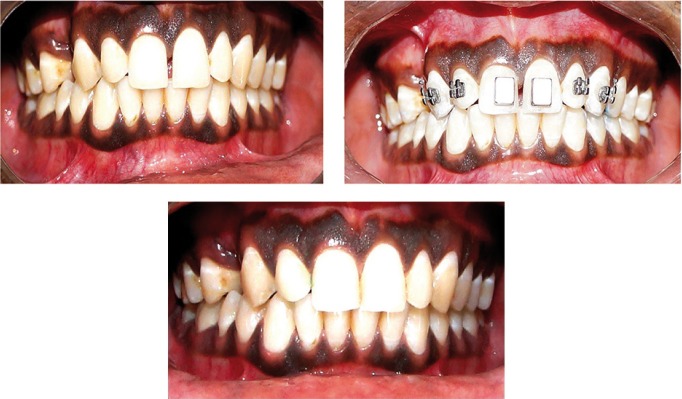
Pre and post treatment intra oral photographs with bonded magnets.

Intra oral photographs of subjects in Group 1 treated with magnets were shown in figure 2. The mean duration of treatment time to close the midline diastema was over all: 13.57 days in the Ne2Fe14B magnets group, 78.20 days in the conventional fixed therapy group ( Table 3) showing a wide variation in the rate of space closure between individuals. The difference in the amount of space closure between the two groups was very large and significant *p*=0.004. Mean change in space closure in 2 groups was shown ( Table 4). Mean change in maxillary incisal inclination (u1-pp0) in degrees and mean change in max incisal angulation to bicondylar line in degrees was also recorded.

**Table 3:**
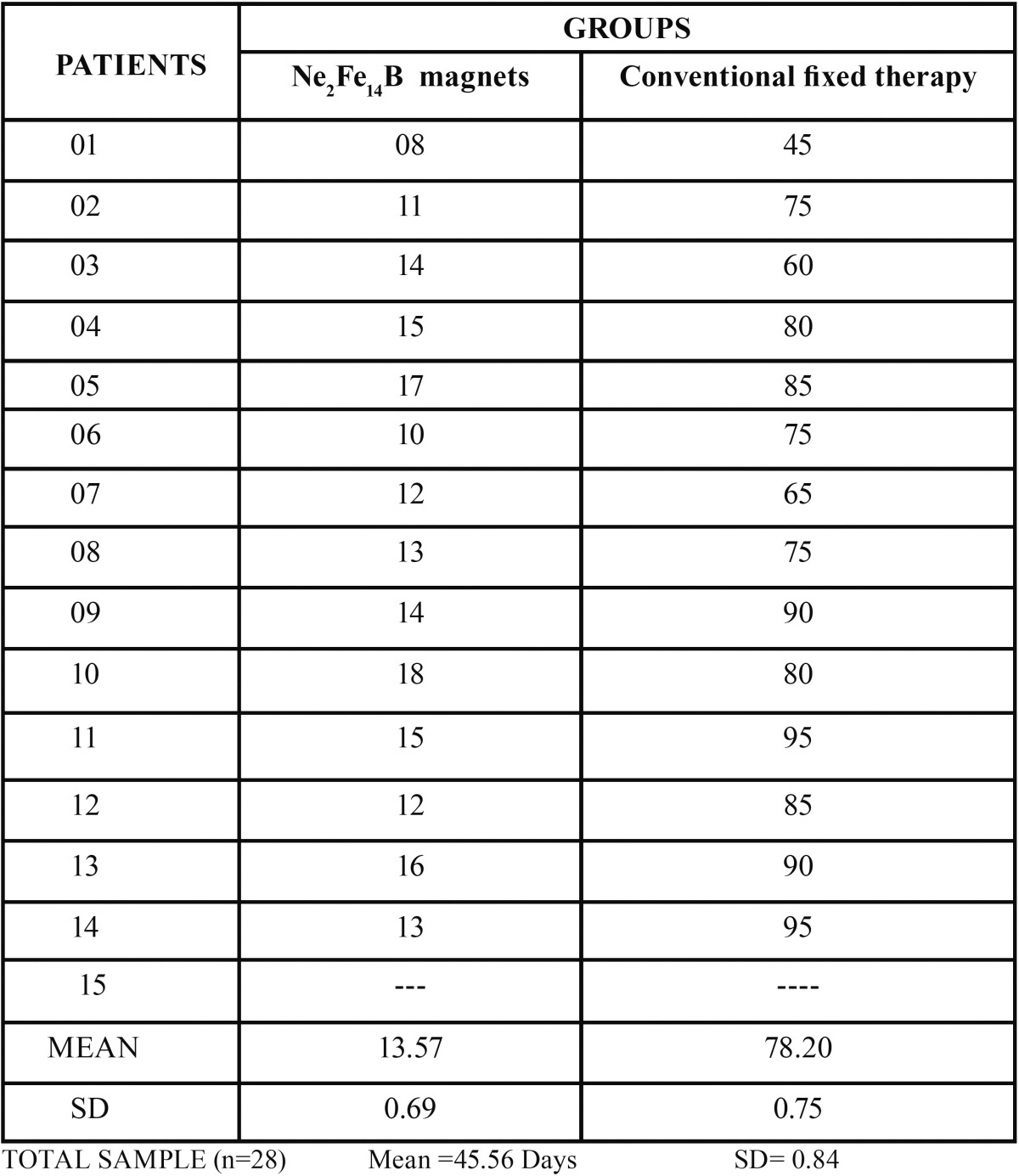
Mean time for space closure in days.

**Table 4:**
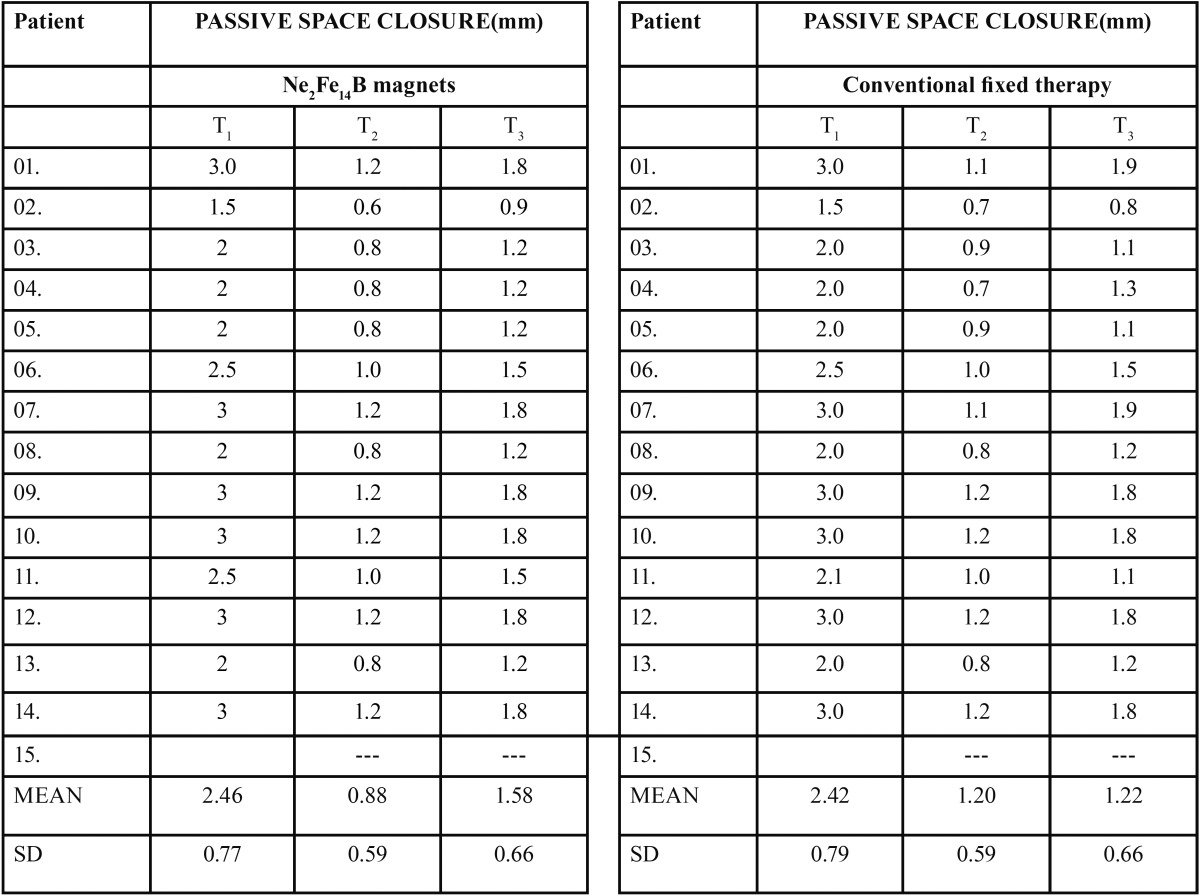
Mean change in space closure.

## Discussion

The relationship between malocclusion and facial form has been a focus of orthodontists since early 20th century (3). The practitioner must consider the contributing factors before determining the optimal treatment. These include normal growth and development, tooth size discrepancies, excessive incisor vertical overlap of different causes, mesiodistal and labiolingual incisor angulation, generalized spacing and pathological conditions. Fixed appliance therapy is one of the most widely used treatment modalities in orthodontic practice. The transition from standard edgewise to pre adjusted appliances had allowed orthodontists to treat patients efficiently and with consistent quality of results (10).

According to epidemiological investigations the prevalence of median diastemas is high in children, decreases dramatically between 9 and 11 years of age and continues as a gradual decrease up to 15 years of age . Diastemas based on tooth-size discrepancy are most amenable to restorative and prosthetic solutions. The most appropriate treatment often requires orthodontically closing the midline diastema. The ideal treatment should seek to manage not only the diastema in question but also the cause behind it to achieve a stable result (11).

Many treatment modalities are used in treating midline diastemas, but fewer studies were conducted on treatment of midline diastema by using magnets. Over the last two decades magnets have been used in orthodontics and attempts have been made to evaluate the biological implications of magnets during clinical application. Use of magnetic forces, though not common, opened new horizons in the field of orthodontic treatment and biomechanics (6). Magnetic therapy (Magnetotherapy) is inexpensive, non-toxic way of treatment. Magnetic force systems are popularly used for relocating impacted teeth, expansion of arch, distalization of molars, intrusion of posterior teeth in open bite cases (6), class II correction with functional appliances, closure of midline diastema.

The first reported use of magnetic force to move teeth was in 1977 when Kawata and Takeda described a technique of using Co-Cr-Fe alloy magnetic brackets. It was concluded that this new magnetic orthodontic tooth movement system was useful not only for space closure but also for derotation and canine retraction. In a study conducted on magnets to evaluate force level, concluded that low force is applied on the tooth and periodontium (5). Magnets tend to attract each other to attain the maximal contact area (5). This property makes teeth upright or rotates them. This requires very accurate positioning of magnets on the teeth so that when incisors touch each other, the magnets also make contact each other. When the magnets are placed too far distally, the incisors make contact first and rotate.

It was found that PEMF of 15Hz create static magnetic rate of tooth movement of about 3mm per month. Also there is absence of classic lag phase initiation of tooth movement. This is explained by the fact that the presence of magnetic field had induced multipotential stem cells to differentiate more rapidly into active osteoclasts, thereby increase in the rate of bone resorption and hence tooth movement (12).

“Darendeliler and Co-workers (6) analyzed the force system diagrams produced by small attracting NdFeB magnets to determine, 1) whether the force levels were sufficient to induce tooth movement, 2) the effect of magnet morphology on force characteristics and, 3) the most appropriate magnet dimensions that could be utilized for this application. He concluded that select range of magnet configurations exhibited suitable and reliable attractive forces and therefore could be advocated for prescribed clinical application (13). In a study on 7-week-old Wistar rats suggested that the PEMF (pulsed electromagnetic field)-induced vibration may enhance the effect of mechanical and magnetic forces on tooth movement.”

Bondemark and Kurol conducted extensive studies on recycling of rare earth magnets used in orthodontics (14). They concluded that autoclaving does not affect the biocompatibility & force stability of the magnets. Since the force stability of Sm-Co magnets has been proven to be stable after a recycling procedure (the force magnitude was decreased in range 0% to 3.5%). It is thus possible from both the biocompatibility and force stability view to reuse magnets, provided that there is no mechanical distortion in the force system. The autoclave heat of 135°C is acceptable since the Curie tempe-rature for Ne2Fe14B magnets is 300°C.

The results of our study however differ with previous studies in the rapidity of alignment, duration of passive space closure and maxillary incisal inclination. The difference could be explained by the variability of components bonded on to the tooth surface (Ne2Fe14B magnets and 0.018 MBT brackets AO type) with their characteristic prescriptions, variability in arch form, arch wire sequence and final working arch wires. From the study conducted, it was obvious in Group 1 subjects (Ne2Fe14B magnets)–cases were treated in less duration of time i.e., 8-18 days with a mean of 13.57 days. The difference in reduction of treatment duration of closure of midline diastemas was 64.63 days and difference in mean space closure between T1-T2 is 1.58 mm and T2-T3 is 0.70 mm.

In comparison, Group 2 subjects treated in more duration of time i.e., 45-95 days with a mean of 78.92 days. The data derived concluded the difference in mean space closure between T1-T2 as 1.22 mm and T2-T3 as 1.20 mm. A mean change in space closure by 3.0 mm and a mean change in maxillary inclination to bicondylar line as 3.90 and a mean change in maxillary inclination as 2 0 (15,16). It was observed the reduction in mean values, a minor change in maxillary incisal inclination in Group 1 subjects (1.85 0) when compared with the change in maxillary incisal inclination in Group 2 subjects (2.50 0). A statistically significant difference was observed in comparison of mean change in maxillary incisal inclination and mean space closure by p value 0.005. The X-rays did not reveal any resorption or damage to the root or periodontium. Rotated, uprighting and even root paralleling was achieved in some cases. No clinical, microscopic and roentgenologic evidence of cytotoxic effects of magnets. Their small size and strong attractive forces allowed to be placed within prostheses without being obtrusive in the mouth.

Since most maxillary midline diastemas recur even after the best-managed treatment, after the space is redistributed permanent retention is necessary (17). A lingually bonded fixed retainer is recommended with good life-long oral hygiene instructions (18). Magnets have been utilized previously for a number of different applications (19) like neodymium-iron-boron micro-magnets as a fixed retainer, which does not hinder oral hygiene, in orthodontic therapy (20,21) even for positioning multi-stranded wire retainers (22). Also consideration should be given for alignment of midline (23). The strength of the study was standardization of clinical variables such as material in composition, dimension, arch wire type, arch wire sequence and inter appointment variables. Consecutive eligible patients were included to minimize confounding variables with method of space closure being the critical difference between treatment groups. Standardization of inclusion criteria, exclusion criteria and the clinical variables is done. Treatment efficiency is the product of many mechanical and biological factors. To summarize, the present study of usage of Ne2Fe14B magnets in closure of midline diastemas, reflected a statistically significant difference in the reduction of mean duration of time and complete space closure when compared with conventional fixed therapy considered as the other (control) group.

## Conclusions

• Ne2Fe14B magnets were more efficient in complete closure of mid line diastema in less duration of time.

• Better 3-dimensional control of the movement of the teeth can be achievable with Ne2Fe14B magnets.

• Ne2Fe14B magnets are most bio-compatible and recyclable with least adverse effects.

• Ne2Fe14B magnets were more efficient in uprighting, root paralleling provided accurate positioning of magnets on the teeth.
